# New Castanospermine Glycoside Analogues Inhibit Breast Cancer Cell Proliferation and Induce Apoptosis without Affecting Normal Cells

**DOI:** 10.1371/journal.pone.0076411

**Published:** 2013-10-04

**Authors:** Ghada Allan, Halima Ouadid-Ahidouch, Elena M. Sanchez-Fernandez, Rocío Risquez-Cuadro, José M. Garcia Fernandez, Carmen Ortiz-Mellet, Ahmed Ahidouch

**Affiliations:** 1 Laboratory of Cellular and Molecular Physiology (EA 4667), SFR CAP-SANTE (FED 4132), UFR of Sciences, UPJV, Amiens, France; 2 Departamento de Química Orgánica, Facultad de Química, Universidad de Sevilla, Sevilla, Spain; 3 Instituto de Investigaciones Químicas (IIQ), CSIC – Universidad de Sevilla, Sevilla, Spain; 4 Department of Biology, Faculty of Sciences, University Ibn Zohr, Agadir, Morocco; The University of Hong Kong, China

## Abstract

sp^2^-Iminosugar-type castanospermine analogues have been shown to exhibit anti-tumor activity. However, their effects on cell proliferation and apoptosis and the molecular mechanism at play are not fully understood. Here, we investigated the effect of two representatives, namely the pseudo-*S*- and *C*-octyl glycoside 2-oxa-3-oxocastanospermine derivatives SO-OCS and CO-OCS, on MCF-7 and MDA-MB-231 breast cancer and MCF-10A mammary normal cell lines. We found that SO-OCS and CO-OCS inhibited breast cancer cell viability in a concentration- and time-dependent manner. This effect is specific to breast cancer cells as both molecules had no impact on normal MCF-10A cell proliferation. Both drugs induced a cell cycle arrest. CO-OCS arrested cell cycle at G1 and G2/M in MCF-7 and MDA-MB-231cells respectively. In MCF-7 cells, the G1 arrest is associated with a reduction of CDK4 (cyclin-dependent kinase 4), cyclin D1 and cyclin E expression, pRb phosphorylation, and an overexpression of p21^Waf1/Cip1^. In MDA-MB-231 cells, CO-OCS reduced CDK1 but not cyclin B1 expression. SO-OCS accumulated cells in G2/M in both cell lines and this blockade was accompanied by a decrease of CDK1, but not cyclin B1 expression. Furthermore, both drugs induced apoptosis as demonstrated by the increased percentage of annexin V positive cells and Bax/Bcl-2 ratio. Interestingly, in normal MCF-10A cells the two drugs failed to modify cell proliferation, cell cycle progression, cyclins, or CDKs expression. These results demonstrate that the effect of CO-OCS and SO-OCS is triggered by both cell cycle arrest and apoptosis, suggesting that these castanospermine analogues may constitute potential anti-cancer agents against breast cancer.

## Introduction

Cancer development is often due to perturbations in the cell cycle that lead to unlimited proliferation and confers apoptosis resistance to cancer cells [1], [2]. The progression of cells through the cell cycle is exerted by cyclin, cyclin-dependent kinases (CDKs), and CDK inhibitors (CKIs). The cyclin-CDK complexes govern a progression of the events that lead cells from a resting state (G0) to growth phase (G1), DNA replication phase (S), and finally to cell division (M). Cyclin D and cyclin E, in association with CDK4 and CDK6, contribute to the G1/S transition, whereas inhibition of the kinase activity of cyclin/CDK complex is mediated by several CKIs, including p21^waf1/cip1^ and p27^kip1^ [3]. Cyclins A and B (along with Cdc2) are important for entry of the cells into the M phase [4]. Aberrantly expressed cell cycle-related cyclins are highly associated with breast cancer [5]. Indeed, cyclin D1 is overexpressed in approximately 50% of breast cancers and is associated with an aggressive tumor phenotype and specific types of p53 [6], [7], [8]. Cyclin E is overexpressed in 30% of breast tumors [9]. Moreover, overexpression of cyclin B1 has also been reported in various human tumors, including breast cancer [10].

Defective apoptosis represents a major causative factor in the development and progression of cancer. Indeed, the majority of chemotherapeutic agents act through the apoptotic pathway to induce cancer cell death. Moreover, resistance to chemotherapeutic strategies seems to be due to alterations in the apoptotic pathway of cancer cells [11]. A factor which is consistently implicated in apoptosis is one of the Bcl2 extended family members [12]. Thus, the anti-apoptotic protein Bcl-2 is expressed in 25% to 50% of breast cancer [13]. Bcl-2 expression has been cited as a favorable prognostic marker [14] and its expression has been shown to be associated with improved survival among patients with breast cancer [14], [15], [16].

Protein glycosylation starts in the endoplasmic reticulum (ER) with a series of steps conserved in the majority of eukaryotes [17], [18]. Glycosidases (I and II) act to sequentially remove the glucose residues from the *N*-glycan in the ER [17], [18]. In a concerted manner, Glucosidases I and II along with lectin chaperones calnexin and calreticulin participate to the ER-retention of nascent glycoproteins until they are properly folded and thus preventing their aggregation [19]. Polypeptides that succeed in reaching their mature conformation are released from the lectin-proteins as soon as their folding process is completed [20], [21], and the unfolded and or misfolded glycoproteins are delivered to cytosolic degradation mediated by the ubiquitin-proteasome pathway, a process known as an ER-associated degradation (ERAD) [22], [23], [24]. Moreover, accumulation of unfolded proteins in the ER induces a condition known as ER stress that in turn, triggers the increased expression of BiP and other chaperones, a phenomenon termed “unfolded protein response” (UPR) [25]. The increased synthesis of ER chaperones, which serve to correct protein misfolding, occurs concomitantly with a marked decrease in the rate of overall protein synthesis [26]. and along with cell arrest in G1 phase of the cell cycle [27], [28]. Inhibition of protein synthesis lowers the overall rate of protein traffic into the ER, thus limiting damage to this organelle. However, If ER stress persists; cells activate mechanisms that result in cell death. Aberrant glycosylation of glycoproteins and glycolipids is a typical molecular feature of malignant cells. For instance, in breast cancer deregulation of the enzymes involved in N-glycosylation of proteins leads to massive formation of polylactosamine chains. These polysaccharides are involved in tumor progression, including proliferation, migration, invasion and angiogenesis [29].

Most efforts up to now have focused in the use of iminosugar inhibitors of the neutral α-glucosidases I and II or α-mannosidase II, such as 1-deoxynojirimycin and castanospermine or swainsonine. A significant reduction in tumor progression and metastasis has been observed in several cases *in vitro* and *in vivo* [30], [31], [32], [33], [34], although, the mechanisms underlying the anti-proliferative action of these substances remain unknown. A new generation of iminosugar-type glycosidase inhibitors termed sp^2^-iminosugars was conceived [35], [36]. Contrary to most classical iminosugars, sp^2^-iminosugars present more affinity towards human glycosidases [37], [38], [39], [40]. Recently, the sp^2^-iminosugar-type 2-oxa-3-oxocastanospermine *N*-, *S*-, and *C*-octyl glycoside derivatives were identified as selective inhibitors of neutral α-glucosidases and evaluated for their anti-growth activity in MCF-7 breast cancer cell line [41]. Whereas the *N*-glycoside NO-OCS had only a modest effect, the *S*- and *C*-glycoside congeners SO-OCS and CO-OCS showed a significant anti-proliferative effect with a concomitant increased in cell mortality [41].

Herein, we report a study on the effect of both SO-OCS and CO-OCS in cell proliferation and apoptosis on MCF-7 and MDA-MB-231 Breast cancer (BC) cells, and normal breast MCF-10A cells and their associated-mechanisms. We demonstrate that both sp^2^-iminosugar derivatives induce cell cycle arrest in BC cell lines. CO-OCS induced an arrest at G0/G1 and G2/M transitions in MCF-7 and MDA-MB-231 cells respectively. In contrast, SO-OCS induced an arrest at G2/M in both cell lines. We identified cyclin D1, cyclin E, CDK1 and 4, pRb and p21^waf1/cip1^ as key potential mediators in CO-OCS and SO-OCS-induced cell cycle arrest. Moreover, both drugs induced apoptosis by regulating Bcl2 and Bax expression. Interestingly, neither SO-OCS nor CO-OCS affected breast normal cell proliferation and apoptosis.

## Materials and Methods

### Drug

**Figure 1:**
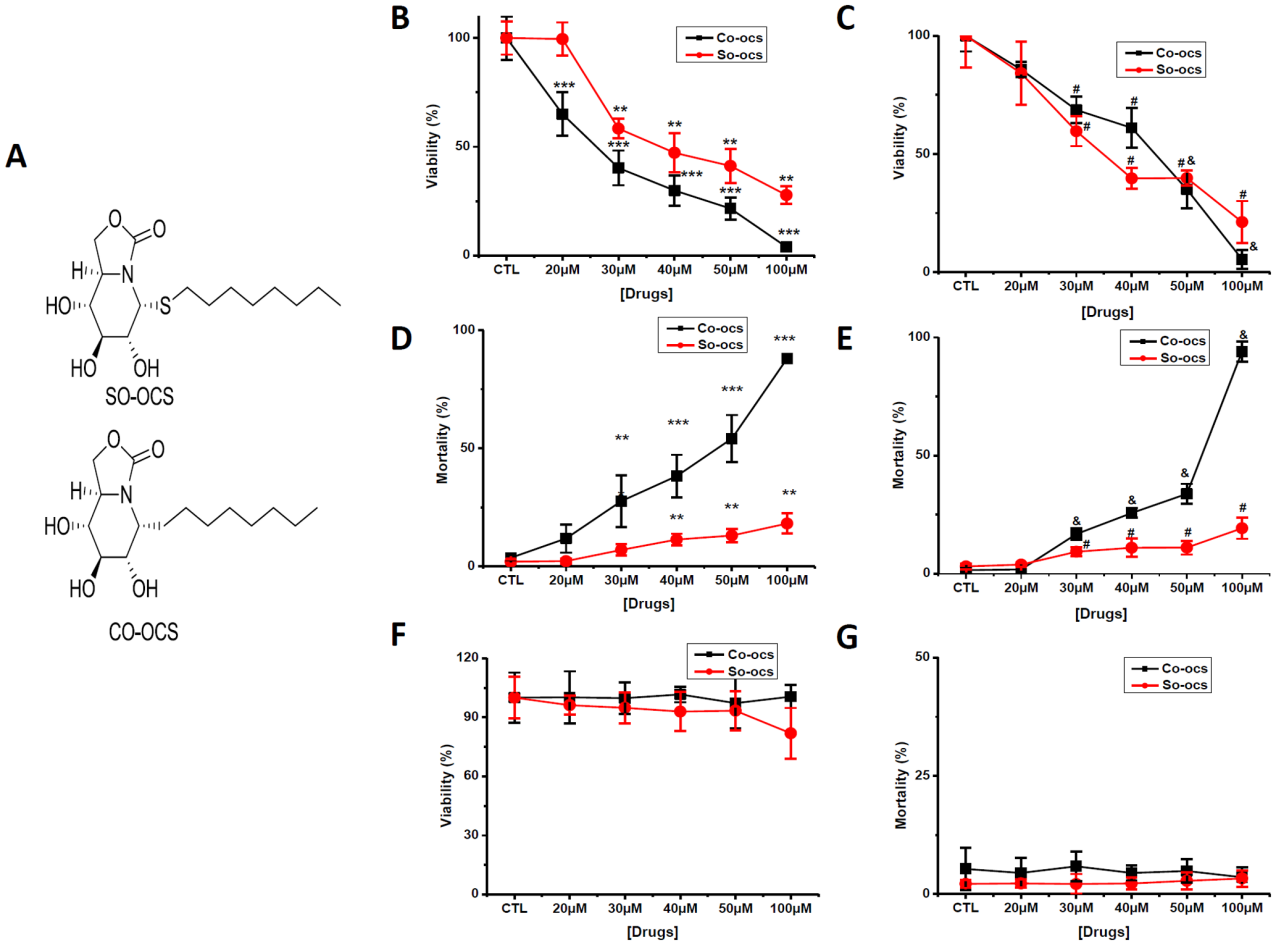
CO-OCS and SO-OCS reduced cell viability and induced mortality in breast cancer cell lines but not in normal ones. Cells are treated with various concentrations of CO-OCS and SO-OCS (0–100 µM) for 72-h. A, Structures of the sp^2^-iminosugar-type *S*-octyl and *C*-octyl 2-oxa-3-oxocastanospermine glycosides SO-OCS and CO-OCS, respectively, evaluated in this work. Inhibitory effects of CO-OCS and SO-OCS on MCF-7 (B) and MDA-MB-231 cells (C). Results were expressed as percentage of the controls, which were arbitrarily assigned 100% viability. CO-OCS and SO-OCS increased cell mortality in MCF-7 (D) and MDA-MB-231 cells (E). Neither CO-OCS nor SO-OCS affects the cell viability (F) and the mortality (G) of MCF 10-A cells. The values indicated are mean ± SD of three independent experiments and analyses by ANOVA test ^*^ *p*<0.05, **p<0.01, ***p<0.001. compared with control group

The 2-oxa-3-oxocastanospermine pseudo-*S*- and *C*-octyl glycosides SO-OCS and CO-OCS (Fig. 1A) were synthesized from acetylated pseudo-glycosyl donor precursors bearing a psudo-anomeric acetoxy (for SO-OCS) or fluoro group (for CO-OCS) by *S*-glycosylation with octanethiol or *C*-glycosylation with trio-ctylaluminum, respectively, following the procedures previously reported [41]. The compounds were solved in DMSO, before storage at –20°C.

### Cell culture

MCF-7 and MDA-MB-231 BC cells were grown in Eagle’s minimum essential medium (EMEM), supplemented with 5% fetal calf serum (Lonza, Levallois-Perret, France), 2 mM L-glutamine, and 0.06% HEPES. The immortalized human mammary epithelial cell line MCF-10A was cultured in complete MCF-10A growth medium, composed of Dulbecco’s modified Eagle’s medium/nutrient mixture F12 (DMEM/F12) supplemented with 5% fetal calf serum, 20 ng/ml epidermal growth factor, 10 mg/ml insulin, 0.5 mg/ml hydrocortisone, and 100 ng/ml cholera toxin (Sigma-Aldrich, St-Quentin Fallavier, France). All cell lines were grown in a 5% CO_2_-humidified incubator at 37 °C.

### Cell viability and mortality

Cells were grown in six petri-dishes (Ø 35 cm) for 2 days, then incubated with SO-COS or CO-COS at different concentrations for 3 days (20, 30, 40, 50, 100 µM). The cells viability and mortality were then assessed by Trypan Bleu assay 72-h after treatment. Cells were harvested by trypsinization and diluted in trypan Blue solution. Cells viability and mortality were assessed using the standard Malassez cell method. Cell counts were performed six times (in a blind manner) and the results were expressed as the percentage of cells (alive or dead) counted normalized to control.

### Flow cytometric analysis

After drug treatment for 72-h at a concentration of 40 µM, the cells were removed by trypsinization, washed twice with phosphate-buffered saline (PBS), then pellets were re-suspended in 300 µL PBS/EDTA and fixed with 700 µL absolute ethanol at −20°C for one day. The cells were treated with ribonuclease (RNA-ease) at concentration 10 µg/ml for 30 min at room temperature and stained with propidium-iodide (PI), (Sigma-Aldrich). The percentage of cells in different phases was measured by flow cytometry with Accuri® and cyflogic system.

### Western Blotting analysis

Whole-cell lysates were prepared with 1% sodium dodecyl sulfate and a protease inhibitor cocktail (Sigma-Aldrich). Proteins were separated by denaturing SDS–PAGE and transferred onto nitrocellulose membranes [42]. The primary antibodies used were: anti-CDK2 (1∶2,000), anti-CDK4 (1∶1,200), anti-cyclin E (1∶1,200, Santa Cruz Biotechnology, Inc., Heidelberg, Germany), anti-cyclin D1 and anti-p21^Waf1/Cip1^ (1:500, Cell Signalling Tech., Danvers, MA), β-actin (used as internal standard, Santa Cruz Biotechnology), anti- protein pRb (1∶1000), phospho-pRb (1∶500), p27^kip1^ (1∶1000), anti-Bcl-2 (1∶1,000), anti-CDK1(cdc2)(1∶1,000), and cyclin B1(1∶2,000) (Cell Signaling Tech), anti-Bax (1∶1,000) (BD Biosciences). Antibodies are followed by secondary antibodies coupled to horseradish peroxidase. Bands were detected using an enhanced chemiluminescence kit (GE Healthcare, Saclay, France) and quantified using the densitometric analysis option in the Bio-Rad image acquisition system (Bio-Rad Laboratories).

### Quantitative apoptosis analysis

Apoptotic cells were detected by performing FITC annexin V apoptosis detection Kit II staining (BD pharmingen). After 72-h of incubation with SO-OCS or CO-OCS at 40 µM, cells were collected, washed twice in ice-cold PBS and re-suspended in 1x binding buffer. FITC annexin V and propidium iodide (PI) were added to the cell preparation and incubated for 15 min at room temperature in the dark. Binding buffer was added to each tube and then the samples were analyzed by flow cytometry (Accuri®).

### Statistical analysis

Results were expressed as means ± SD, statistical analysis of the data were performed using two-tailed unpaired (between two groups) or a one way analysis of variance (ANOVA, among three or more groups). We compared the mean of each group with that of the control. Differences were considered significant when *p* value is < 0.05.

## Results

### CO-OCS and SO-OCS decrease breast cancer cell growth but not affect proliferation of normal breast cells

We investigated the sensitivity of breast cancer and breast normal cell lines to the sp^2^-iminosugar glycosides CO-OCS and SO-OCS using the Trypan Blue exclusion assay. Both CO-OCS and SO-OCS inhibited cell viability in a dose dependent manner. IC_50_ values, determined after treatment in complete medium for 72-h, are shown in Table 1. MCF-7 cells were more sensitive to the *C*-glycoside CO-OCS than to the *S*-glycoside SO-OCS (Figure 1B), while MDA-MB-231 cells showed similar sensitivity to both compounds (Figure 1C). We also estimated cell mortality under the same conditions. CO-OCS increased cell mortality from a concentration of 30 µM for MCF-7 (Figure 1D) and MDA-MB-231 cells (Figure 1E). SO-OCS induced less mortality than CO-OCS in both cancer cell lines (less than 20% at 100 µM). Interestingly, CO-OCS as well as SO-OCS failed to affect both viability and mortality of normal MCF-10A cells at concentrations up to 100 µM (Figure 1F-G).

**Table 1:** IC_50_ value determined after treatment with the *C*-glycoside CO-OCS and the S-glycoside SO-OCS in complete medium for 72-h.

	MCF-7	MDA-MB-231
CO-OCS	26 µM	44 µM
SO-OCS	37 µM	35 µM

### CO-OCS and SO-OCS reduce cell proliferation by inducing cell cycle arrest

**Figure 2:**
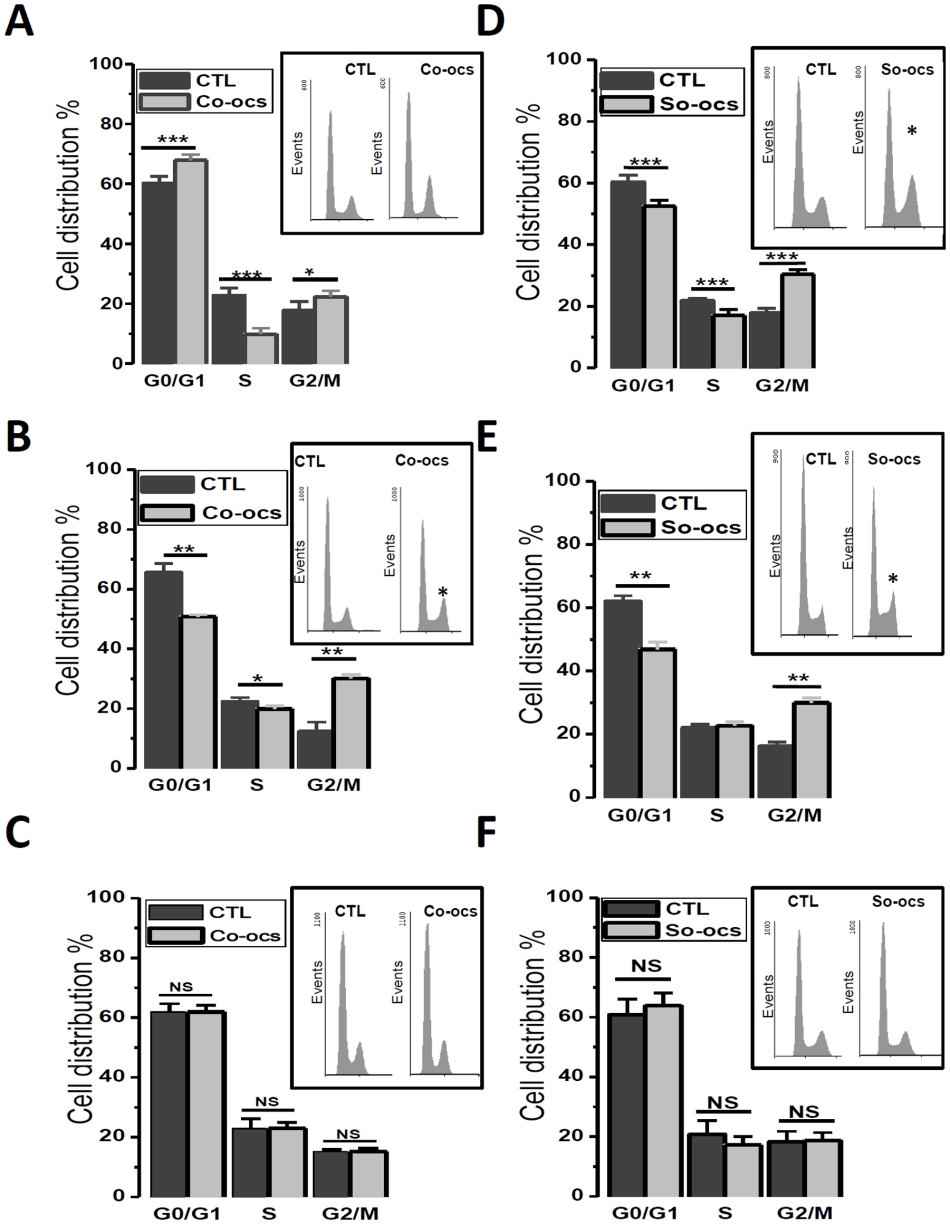
CO-OCS and SO-OCS arrested cell cycle at specific phases in breast cancer cell lines but not in normal cell line. Cells were treated with CO-OCS or SO-OCS (40 µM) for 72-h. Thereafter, cells were collected by trypsinization, stained with propidium iodide and the DNA content was measured by flow cytometry. The percentage of cells within G0/G1, S, G2/M, were determined. CO-OCS accumulated cells in the G0/G1 and G2/M phases in MCF-7cells (A), and in G2/M phase in MDA-MB-231 cells (B). However, SO-OCS accumulated cells in G2/M phase in both MCF-7 and MDA-MB-231 cells (D, E). No synchronization was observed in MCF-10A cells (C, F). The results represent means ± SD of three independent experiments. *Inserts*: traces representing distribution of the cells in the cell cycle. Asterisks denote statistical significance as compared to control cells ^*^ *p*<0.05, ^**^ *p*<0.01, ^***^ *p*<0.001.

To investigate the mechanism accounting for the anti-proliferative activity of CO-OCS and SO-OCS, the cell cycle distribution was analyzed in both normal and cancer breast cell lines. All cells were treated for 72-h with the corresponding sp^2^-iminosugar glycoside at 40 µM. As determined by flow cytometry, MCF-7 treatment by CO-OCS resulted in a clear increase in cell percentage in G1 phase and G2/M with a concomitant decrease in the percentage of cells in the S phase when compared with control (Figure 2A, *p*<0.001). The same treatment accumulated cells in G2/M in MDA-MB-231 cells, with a decrease of cells in G1 and S phases when compared to control (Figure 2B, *p*<0.001). Otherwise, the thio-glycoside SO-OCS induced accumulation of cells in G2/M with a decrease of cells in G1 and S phases in both MCF-7 and MDA-MB-231 cells (Figure 2C, D, *p*<0.001). Table 2 shows the percentage of cells in the cell cycle under different conditions. Similar experiments were performed on normal MCF-10 A cells and, interestingly, no significant effect was observed on cell cycle distribution after 72-h of treatment with CO-OCS (Figure 2E) or SO-OCS (Figure 2F). These data are consistent with an induction of cell arrest in G1 and G2/M by CO-OCS and SO-OCS, respectively.

**Table 2:** Percentage of normal and malignant breast cells treated with CO-OCS and SO-OCS in G0/G1, S, and G2/M phases of the cell cycle.

	phase	CTL	CO-OCS	CTL	SO-OCS
	G0/G1	60.25±2.17	67.86±1.9	60.33±2.2	52.5±1.8
**MCF-7**	S	22.8±2.3	9.8±2	20.7±0.75	17.07±1.9
	G2/M	17.8±2.8	22±2	18.75±2.5	30.38±1.4
	G0/G1	65.46±3.1	50.76±0.62	61.96±1.8	46.89±2.2
**MDA-MB-231**	S	22.25±1.4	19.88±1.05	21.95±1.16	22.74±1.27
	G2/M	12.30±3.2	30.12±1.3	16.17±1.42	30.01±1.45
	G0/G1	61.87±2.8	61.88±2.3	60.92±5	63.95±4
**MCF-10A**	S	22.92±3.28	22.9±1.87	20.79±4.6	17.23±2.8
	G2/M	15.18±0.68	15.12±1.18	18.27±3.5	18.79±2.6

### CO-OCS and SO-OCS regulate the expression of cell cycle proteins involved in G1 and G2/M phases

To elucidate the molecular mechanisms involved in the observed cell cycle alterations, we investigated the effect of CO-OCS and SO-OCS on the expression of cell cycle protein actors. We focused on the proteins that play a crucial role in G1 and G2/M phases. It is known that cyclins D1, E and CDK4/CDK2 and pRb proteins play an important role in controlling the G1 progression and G1?S transition of the cell cycle [43] and cyclin B1 and Cdc42 (CDK1) in G2/M [44], [45]. Compounds that reduce the expression and or activity of these proteins could be expected to reduce breast cancer cell proliferation.

**Figure 3:**
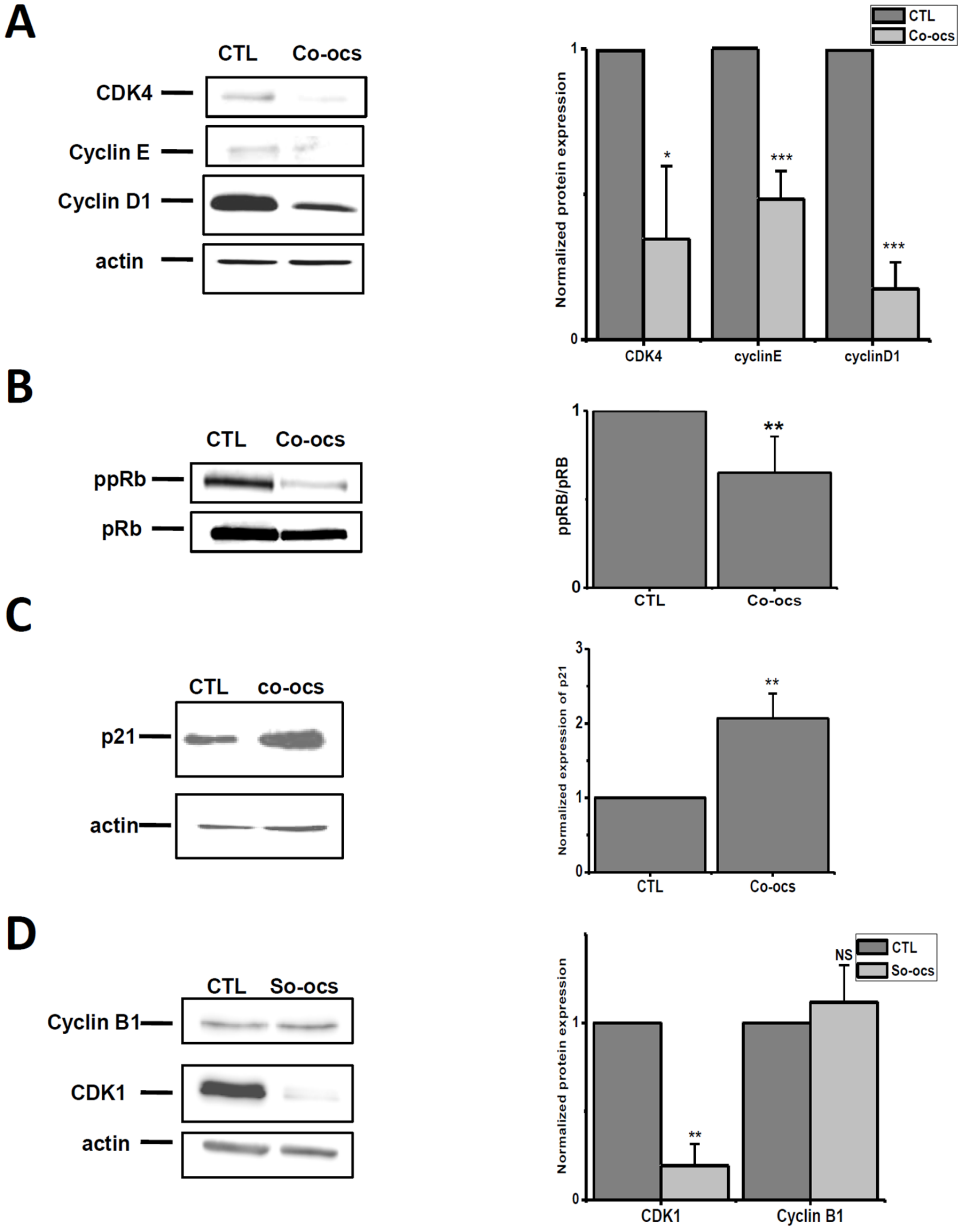
Effect of CO-OCS and SO-OCS on the expression of G1 and G2/M key proteins in MCF-7 cells. MCF-7 cells were treated with CO-OCS and SO-OCS (40 µM) for 72h. Cells were harvested at the indicated times and lysed in RIPA buffer. Cell lysates were analyzed by Western blotting using primary antibodies directed against CDK4, cyclin D1 (A), cyclin E (A), p21^Waf1/Cip1^ (C), and cyclin B1 proteins (D). B, Effect of CO-OCS on phosphorylated Rb protein (ppRb) vs Rb protein (pRb). For each protein, a representative immunoblot of three independent experiments is shown (left panel). Protein levels were quantified and normalized to actin or pRb (right panel). The indicated values are the mean ± SD of three independent experiments (right panel). ^*^p<0.05, ^**^ p<0.01, ^***^ p<0.001.

**Figure 4:**
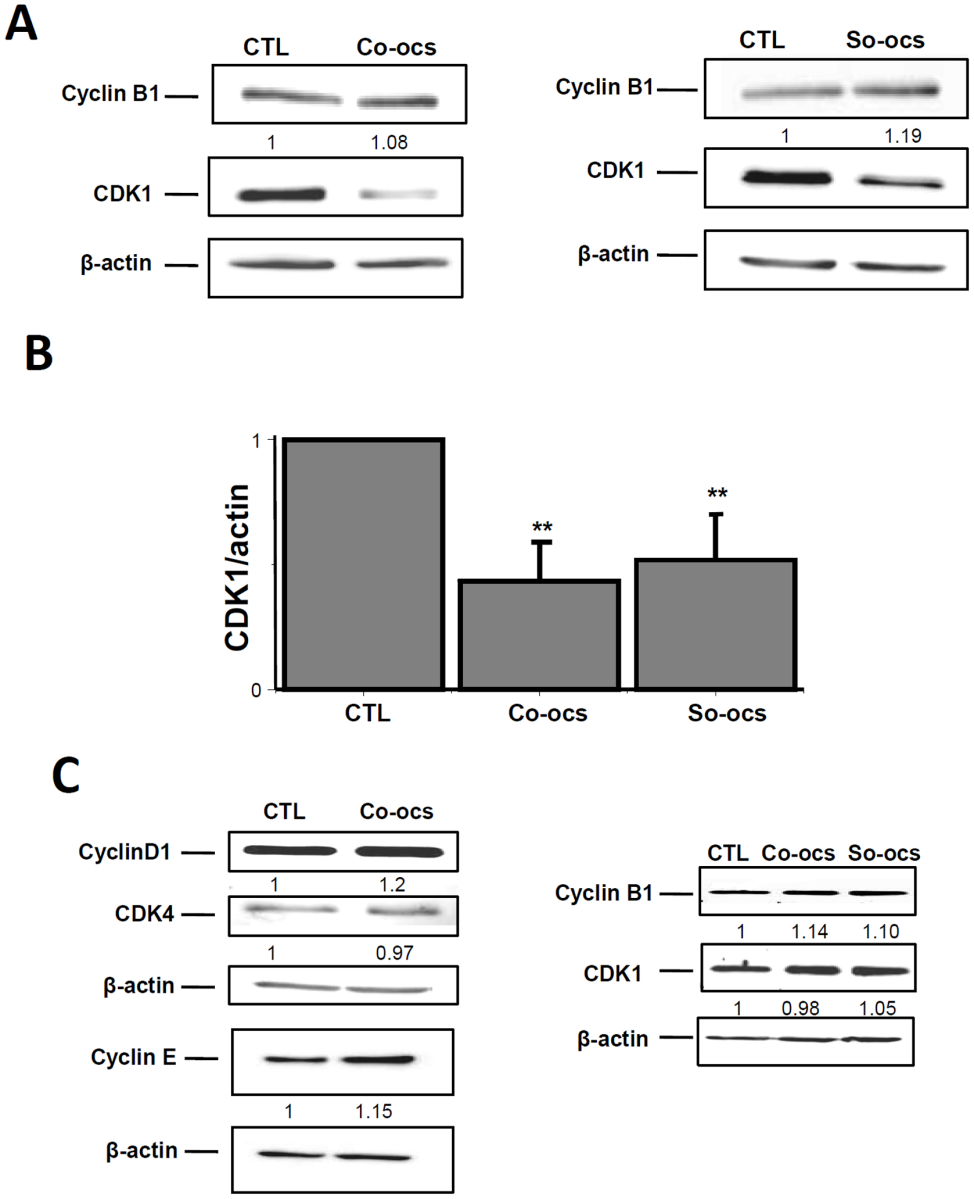
Effect of CO-OCS and SO-OCS on the expression of G1 and G2/M key proteins in MDA-MB-231 and MCF-10A cells. MDA-MB-231 cells were incubated with CO-OCS or SO-OCS (40 µM) for 72-h. Immunoblots showed a decrease in CDK1 (cdc2) expression, but not in cyclin B1 expression (A). B, Protein levels were quantified and normalized to actin. C, Representative immunoblots of three independent experiments in MCF-10 A cells. Both CO-OCS and SO-OCS were without effect on the expression of CDK4, CDK1 (cdc2), cyclin D1, cyclin E and cyclin B1 protein. The results represent mean ± SD of three independent experiments. ^**^ *p*<0.01.

Cells were treated with the sp^2^-iminosugars at 40 µM concentration for 72-h. They were then harvested by RIPA and the cell cycle proteins levels were analyzed by western blotting. In MCF-7 cells, the pseudo-*C*-glycoside CO-OCS decreased the expression of cyclin D1, cyclin E, and CDK4 by 82%, 51% and 64% respectively (Figure 3A). Moreover, it reduced phopshorylation of pRb by 46% (Figure 3B) and increased the expression of p21^Waf1/Cip1^ by 2.2 folds over control (Figure 3C). The pseudo-*S*-glycoside SO-OCS decreased CDK1 expression by 81%, but failed to affect cyclin B1 expression (Figure 3D). Treatment of MDA-MB-231 cells by CO-OCS or SO-OCS decreased CDK1 expression by 67% and 49% respectively, but was without any effect on cyclin B1 expression (Figure 4 A, B). Finally, similar experiments have been performed on MCF-10 A cells and no effect was observed on the expression of CDK4, CDK1, cyclins D1, E, and B1 (Figure 4C).

### CO-OCS induces apoptosis in MCF-7 and MDA-MB-231cell lines, while SO-OCS induces apoptosis only in MCF-7 cells

**Figure 5:**
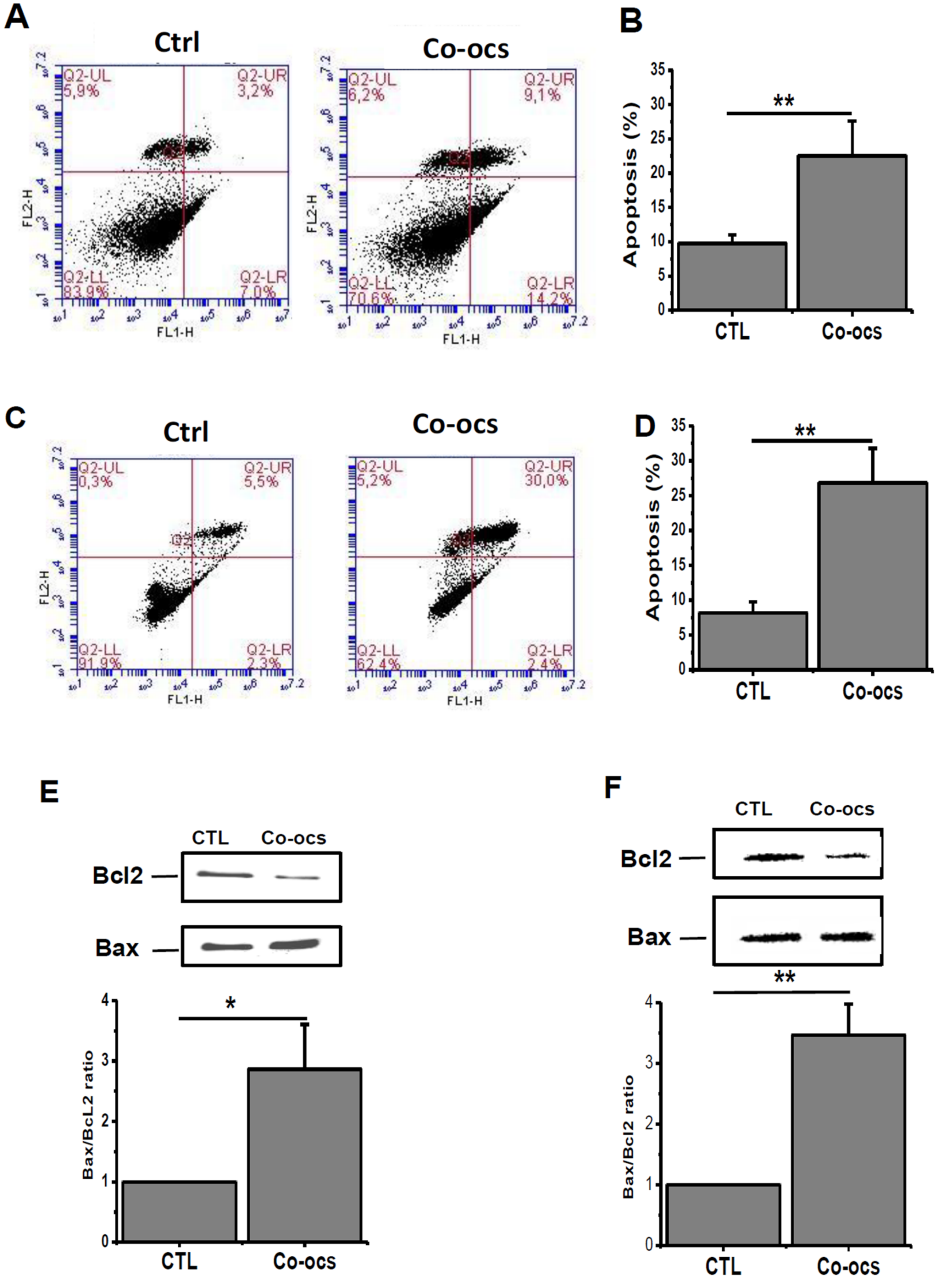
CO-OCS induced apoptosis in MCF-7 and MDA-MB-231 cell lines. Cells were treated with CO-OCS for 72-h and the apoptosis assay was carried out by annexin V staining. CO-OCS (40 µM) induces apoptosis in both MCF-7 (A, B) and MDA-MB-231 cells (C, D). Representative traces of three independent experiments are shown on the left panel. The indicated values are the mean ± SD of three independent experiments (right panel) ^**^ p<0.01. E, F, Treatment with CO-OCS increases the ratio Bax/Bcl2 in MCF-7 (E) and MDA-MB-231 cells (F). All experiments were performed at least three times in independent cell culture. ^*,**^ significantly different form control at *p*<0.05, and 0.01 respectively.

**Figure 6:**
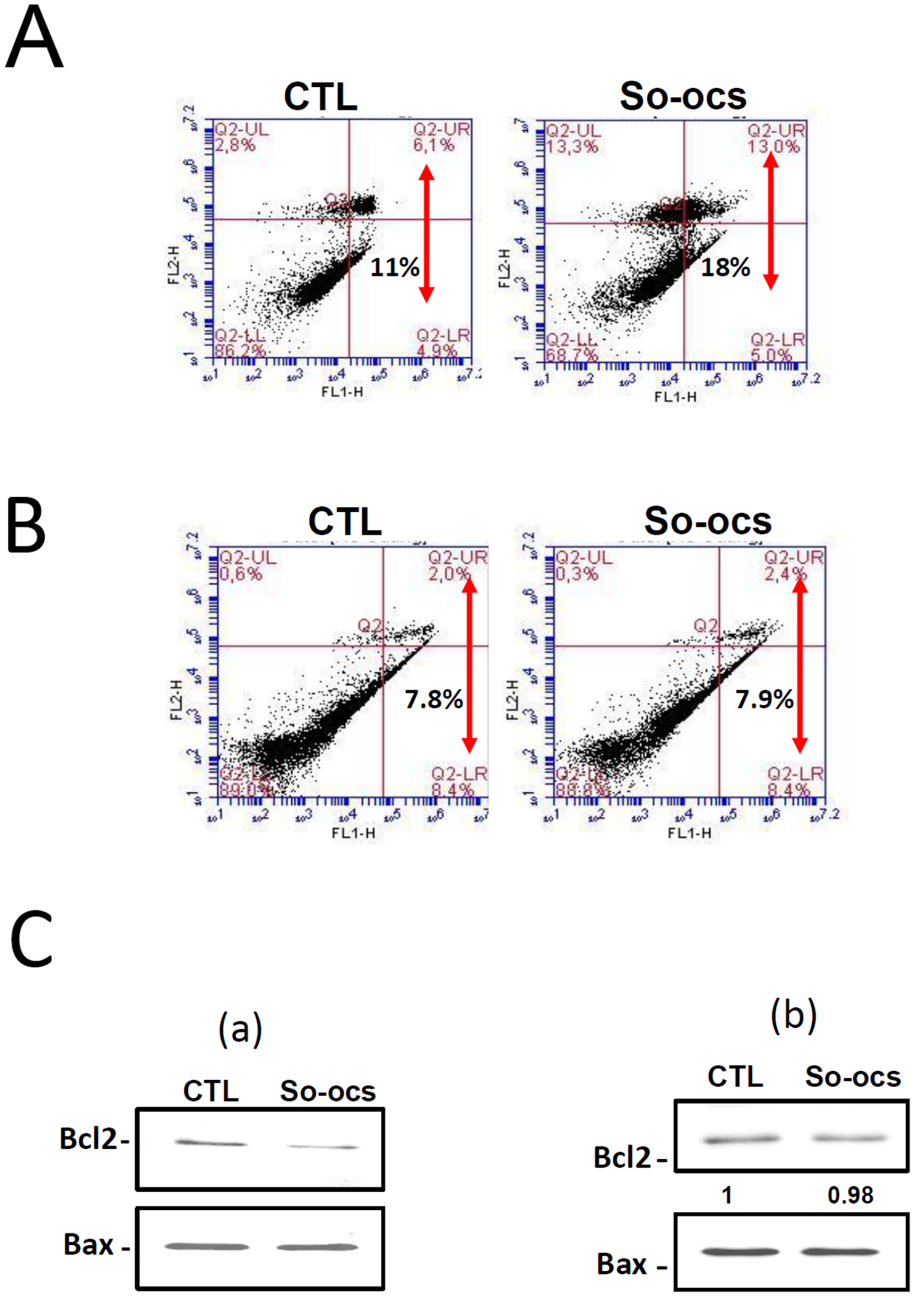
SO-OCS induced apoptosis in MCF-7 cells but not in MDA-MB-231 cells. A Representative apoptosis assay carried out by annexin V staining after 72-h treatment of MCF-7 (A) and MDA-MB-231 cells (B) with SO-OCS at concentration of 40 µM. C, Representative Western blot showing the effect of SO-OCS on Bax and Bcl-2 expression in MCF-7 cells (C-a) and MDA-MB-231 cells (C-b). All experiments were performed at least three times in independent cell cultures. ^*^ Significantly different from control at *p*<0.05.

Since the pseudo-*C*-glycoside CO-OCS induces more cell mortality in BC cell lines than the pseudo-*S*-glycoside analogue SO-OCS, it was of interest to establish whether this mortality is attributed to apoptosis. Both MCF-7 and MDA-MB-231 cells were treated with CO-OCS and SO-OCS (40 µM) for 72-h and subsequently subjected to double staining with annexin V FITC and propidium Iodide (PI) followed by flow cytometry analysis. Only annexin V–stained cells were considered as apoptotic cells. The apoptotic cell percentage increased from 9.74±1.24% to 22.52±5% for MCF-7 cells (Figure 5A, B, *p*<0.01) and from 8.2±1.57% to 26.85±4.9% for MDA-MB-231 cells (Figure 5C, D, *p*<0.01). To confirm this result we quantified the expression of the anti-apoptotic protein Bcl-2 and the pro-apoptotic protein Bax in cells treated with CO-OCS. The Bax to Bcl-2 ratio determines cellular survival or apoptotic cell death [46], [47]. The Bax/Bcl-2 ratio was increased by 2.8±0.7-folds in MCF-7 (Figure 5E, *p*<0.05), and 3.46±0.53-folds in MDA-MB-231 cells (Figure 5F, *p*<0.01) treated by CO-OCS when compared to the control. In contrast, SO-OCS induced apoptosis only in MCF-7 cells. The level of apoptosis increased from 11.95±7.78 to 19.02±9.4 (Figure 6A, *p*<0.05), but it did not cause any considerable apoptotic effect in MDA-MB-231 cells (Figure 6B). Furthermore, the ratios of Bax to Bcl-2 protein expression revealed that cells treated with 40 µM of SO-OCS showed a 1.5±0.19-folds increase in MCF-7 (Figure 6C-a).

We also investigated the effect of SO-OCS and CO-OCS on apoptosis in MCF-10A by flow cytometry using Annexin V, Propidium Iodide double-staining. Neither SO-OCS nor CO-OCS affects apoptosis (Figure S1 A-B). Taken together, these findings demonstrate an important role for the Bcl2 family of proteins in CO-OCS induced apoptotic cell death in cancer cells.

## Discussion

Several studies have explored the anti-tumour effect of the indolizidine alkaloid castanospermine and related iminosugar-type glycosidase inhibitors *in vitro*. Thus, CS inhibits tumor cell invasion and migration [34] and angiogenesis [33], [48]. The administration, *in vivo,* of CS during the early phase of tumor growth in mice delayed the growth of murine and human prostate cancer cells [33] and reduced pulmonary colonization of metastatic murine melanoma cells [31]. The anticancer activity of the iminosugars has been generally ascribed to its ability to inhibit ER and Golgi neutral glycosidases, thereby affecting the biosynthesis of the glycan chains in N-glycoproteins, although the mechanisms at play remain poorly known. The broad range glycosidase inhibitory profile generally exhibited by iminosugars, particularly the simultaneous inhibition of the lysosomal acid glycosidase isoenzymes, hampers their application in the clinics [49]. In a preliminary study [41], we reported the synthesis of CS-related sp^2^-iminosugars with pseudo-glycoside structure as selective inhibitors of neutral α-glucosidases. Notably, the pseudo-*C*- and pseudo-*S*-octyl glycosides CO-OCS and SO-OCS significantly inhibited proliferation of MCF-7 breast cancer cells *in vitro*. Contrary to the parent iminosugar CS, none of these sp^2^-iminosugars affected human lysosomal acid α-glucosydase or intestinal maltase-glucoamylase, which reduces the risk of unwanted secondary effects. Exploring the molecular basis and biochemical routes responsible for the antiproliferative activity of CO-OCS and SO-OCS was, thus, very propitious.

In this study we have investigated the mechanisms operating in the anti-cancer activity induced by the CS-related sp^2^-iminosugar pseudo-*C*- and pseudo-*S*-octyl glycosides CO-OCS and SO-OCS in (BC). We show that CO-OCS and SO-OCS reduce BC cell viability with different sensitivity. The pseudo-*C*-glycoside CO-OCS is more potent in inhibiting non-invasive MCF-7 (IC_50_  =  26 µM) than invasive MDA-MB-231 BC cells (IC_50_  =  44 µM), while the pseudo-*S*-glycoside SO-OCS has similar inhibitory potencies for both cell lines (IC_50_ about 35 µM). Moreover, CO-OCS is more efficient than SO-OCS at inhibiting proliferation of MCF-7 cells, while the two compounds present similar inhibitory potencies against MDA-MB-231 cells.

The sp^2^-iminosugar glycosides CO-OCS and SO-OCS are able to induce cell cycle arrest and apoptosis in triple positive MCF-7 and triple negative MDA-MB-231 cells, while they exert no effect on normal breast MCF-10A cells even at high concentrations. Cyclins and CDKs are the key regulators of the cell cycle G1 phase, the G1/S transition and G2/M phase [50]. Our flow cytometry analysis shows that CO-OCS induces cell cycle arrest at the G0/G1 phase in MCF-7 and G2/M in MDA-MB-231 cells; while SO-OCS induces an arrest in G2/M in both cell lines. The G0/G1 block obtained upon treatment with CO-OCS is due to a reduction in CDK4, cyclin D1 and cyclin E expression, a reduction in pRb phosphorylation and an upregulation of p21^CIP1^expression. Indeed, cyclin D1 plays an important role in controlling the G0/G1 progression and G1/S transition of the cell cycle by activating their cyclin–dependent kinases (CDK4 and CDK2) and cyclin E, which leads to phosphorylation of the retinoblastoma protein (pRb) and, in turn, let the cells to progress through the G1 phase of the cell cycle [51], [52]. The block at G2/M phase induced by the *C*-octyl glycoside CO-OCS in MDA-MB-231 cells and by the *S*-octyl glycoside SO-OCS in the MCF-7 and MDA-MB-231cell lines was accompanied by a decrease of CDK1 (cdc2) expression, without affecting the expression of cyclin B1.

Both CO-OCS and SO-OCS are potent inhibitors of ER neutral α-glycosidase (*K*_i_ 0.87 and 3.4 µM, respectively, for the yeast enzyme). It is well known that the N-glycosylation process participates in the folding of quality control of proteins synthesized via ER [53]and that the inhibition of this process can lead to accumulation of misfolded proteins within the ER that trigger the UPR [54]. The UPR coordinates the induction of ER chaperones with decreased protein synthesis and growth arrest in the G1 phase of the cell cycle which likely serves as a stress-induced response that allows cells to reestablish ER homeostasis [27], [55], [56], [57]. Several studies have demonstrated the cyclin D1 as a crucial downstream in UPR-induced cell cycle arrest. Indeed, unfolded protein response inhibits cyclin D1 translation and expression in mouse NIH3T3 fibroblasts [56], [57]. Loss of cyclin D1 expression was also observed in several types of human cancer cells lines treated with α-glucosidase inhibitors, such as N-methyl-1-deoxynojirimycin (MDNJ) and N- [8-(3-ethynylphenoxy) octyl]-1-deoxynojirimycin [58], [59], or tunicamycin, the classical and the most used inhibitor of N-glycosylation [60], [61]. Moreover, overexpression of cyclin D1 prevented cell-cycle arrest at higher concentrations of tunicamycin, as well as by thapsigargin, indicating a general role for cyclin D1 loss in UPR-induced arrest [56]. However, a recent study, in melanoma cells, has demonstrated the involvement of p27 (increase of expression) rather than cyclin D1 in G1 cell cycle arrest induced by tunicamycin [62] and another study, in human breast cancer cells, showed that knockdown of PERK, results in cell cycle arrest in G2/M phase [63]. Given that ER stress-mediated activation of the UPR occurs in cancer tissues and cell lines [64], [65], [66], we can hypothesized that the observed effects of the CS-related sp^2^-iminosugar glycosides in BC cells cycle, that is the blockade at G1 in MCF-7 and at G2/M in MDA-MB-231 cells by CO-OCS and at G2/M in either of the two BC cell lines by SO-OCS, constitute an adaptive response for survival, although the molecular mechanisms remain to be determined.

CO-OCS and SO-OCS induced cellular mortality that was mainly apoptotic as demonstrated by the increased percentage of annexin V positive cells and Bax/Bcl-2 ratio in MCF-7 and MDA-MB231cells. The *C*-octyl glycoside CO-OCS is more potent than the *S*-octyl glycoside congener SO-OCS, which failed to induce apoptosis in MDA-MB-231 cells. Several members of the proteins that control apoptosis, including the anti-apoptotic protein Bcl2 and the pro-apoptotic protein Bax are expressed in breast carcinoma [67], [68]. Our results show a relationship between the type of apoptosis (precocious or delayed) and the expression of Bcl2 and Bax. CO-OCS increased precocious apoptosis in MCF-7 that was associated with a decrease in the expression of Bcl2 expression and an increase in Bax expression. However, in MDA-MB-23 it induced a delayed apoptosis which is associated with a reduction of Bcl2 but without any significant effect on Bax expression. Similar result was obtained with SO-OCS in MCF-7 cells.

Several studies have proposed the polyhydroxylated alkaloids as apoptotic inducers in cancer cells. Indeed, swainsonine (SW), a natural indolizidine alkaloid with strong Golgi α-mannosidase II inhibitory activity, induces apoptosis in human gastric, lung, and oesophageal squamous cancer cells through the activation of mitochondrial pathway. In human gastric carcinoma cells and rat glioma cells, SW induces apoptosis by involving Ca^2+^ overload and Bcl-2 expression decrease [69], [70]. These authors reported recently that SW treatment induces oesophageal squamous cancer cells apoptosis by up-regulating Bax and down-regulating Bcl-2 expression [71]. The fact that both sp^2^-iminosugars CO-OCS and SO-OCS behave as inhibitors of ER α-glucosidases and also regulate the expression of Bcl2 and Bax supports a relationship between the N-glycoprotein biosynthesis and ER stress-associated apoptotic effect, although alternative mechanistic pathways cannot be discarded. Thus, it is known that a decrease in the expression of CDK1 along with an increase in that of p21^waf1/cip1^ may lead to apoptosis [72]. Since CO-OCS and SO-OCS decrease the expression of CDK1 in both MCF-7 and MDA-MB231 and CO-OCS increases p21^waf1/cip1^ expression in MCF-7 cells, we cannot exclude the involvement of these actors in apoptosis.

In summary, the biological evaluation of sp^2^-iminosugar-type castanospermine analogues led to the identification of the *C*- and *S*-octyl glycosides CO-OCS and SO-OCS as inhibitors of breast cancer cell growth *in vitro*. The two substances inhibit tumor cells proliferation by inducing cell cycle arrest and induce apoptosis, displaying a range of properties of interest in the development of new antitumor agents.

## Supporting Information

Figure S1 A representative apoptosis assay carried out using annexin V staining after 72-h treatment of MCF-10A with 40 µM Co-ocs (B) and 40 µM So-ocs (C). Both Co-ocs and So-ocs failed to induce apoptosis when compared to control conditions (A). Experiments were performed 2 times in 2 independent cell culture conditions.
(TIF)
