# Supplementary figures and images for: New Castanospermine Glycoside Analogues Inhibit Breast Cancer Cell Proliferation and Induce Apoptosis without Affecting Normal Cells
Source: PLoS One. 2013 Oct 4;8(10):e76411. doi: 10.1371/journal.pone.0076411 (PMC3790671; doi:10.1371/journal.pone.0076411)

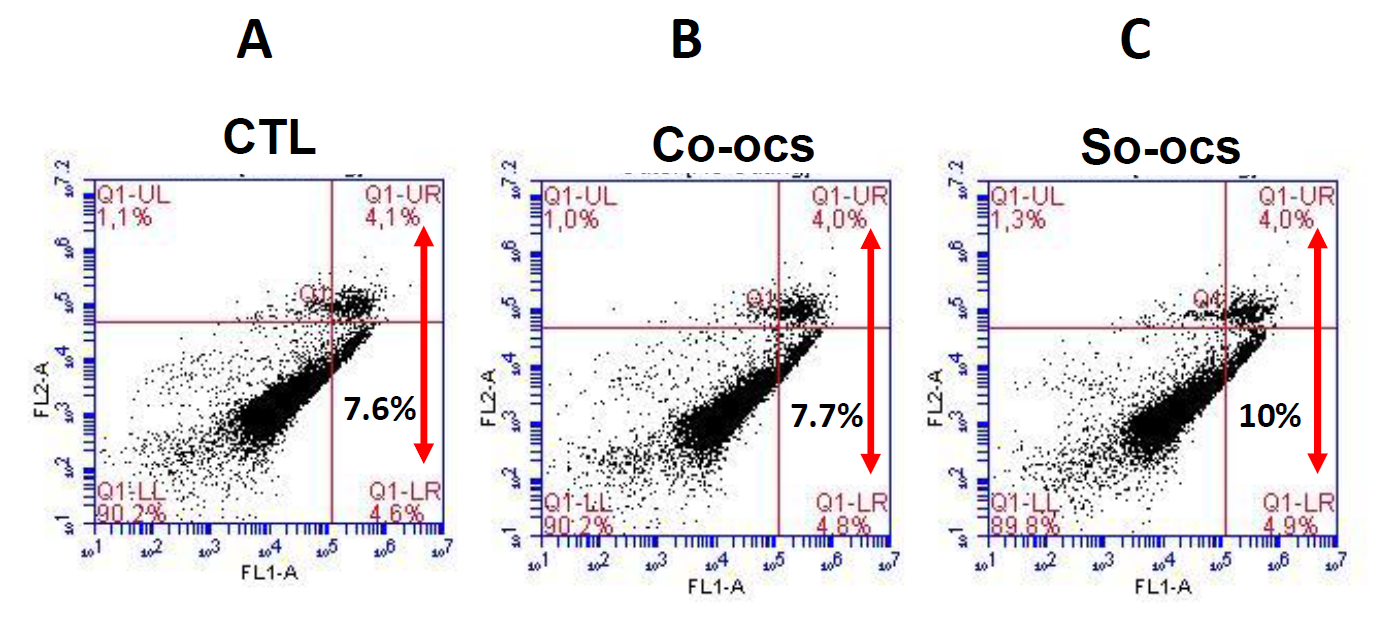

Supplement: Figure S1 — A representative apoptosis assay carried out using annexin V staining after 72-h treatment of MCF-10A with 40 µM Co-ocs (B) and 40 µM So-ocs (C). Both Co-ocs and So-ocs failed to induce apoptosis when compared to control conditions (A). Experiments were performed 2 times in 2 independent cell culture conditions. (TIF) [file pone.0076411.s001.tif]
